# Supplementary material for: Assessment of ADHD Subtypes Using Motion Tracking Recognition Based on Stroop Color–Word Tests
Source: Sensors (Basel). 2024 Jan 5;24(2):323. doi: 10.3390/s24020323 (PMC10818498; doi:10.3390/s24020323)
Supplement: Supplementary file 1 [file sensors-24-00323-s001.zip › sensors-2717072-supplementary.pdf]

Supplementary Materials: Assessment of ADHD subtypes using motion tracking recognition based on Stroop color-word tasks

Chao Li [1,2], David Delgado-Gómez [3], Aaron Sujar [4], Ping Wang [1,2], Marina Martin-Moratinos [1,2], Marcos Bella-Fernández [2,5,6], Antonio Eduardo Masó-Besga [7] and Inmaculada Peñuelas-Calvo [8], Juan Ardoy-Cuadros [9], Paula Hernández-Liebo [10] and Hilario Blasco-Fontecilla [11,12,*]

Analytical Model and Supplementary Results

Correct Rate

For the analysis of the correct rate, the following formula from the linear mixed effects model was used to investigate the variables that affect the correct rate in the SCWT test and taken into account the subtests, the ADHD group, age, medication use and left-handedness:

```
Correct Rate ~ Subtests+ ADHD Group+ Age+ Medication Treatment
+ Left handedness+ (1|Patient ID)
```

Both data sets based on adjusted and unadjusted RT were used in this linear mixed effects modeling. They have very similar results. Table S1 shows the results based on the adjusted RT data.

Table S1. Coefficients, standard errors, t-values, and p-values for correct rate

Variable[1]	Coefficient(95%CI)	SE	t	*p-value*[2]	*Adj.p*[2]
Intercept	93.07 (82.62, 103.53)	5.30	17.57	**<0.001**	**<0.001**
Age	1.18 (0.23, 2.12)	0.48	2.46	**0.01**	**0.04**
ADHD-I	1.86 (-3.60, 7.31)	2.76	0.67	0.50	0.50
Left-Handedness	-3.36 (-12.06, 5.34)	4.41	-0.76	0.45	0.50
Color Test	-1.38 (-4.53, 1.77)	1.59	-0.87	0.39	0.50
Color-Word Test	-4.14 (-7.28, -0.99)	1.59	-2.59	**0.01**	**0.04**
Medication Treatment	-2.52 (-7.73, 2.69)	2.64	-0.96	0.34	0.50
**ICC**					
Adjusted ICC	0.426				
Unadjusted ICC	0.376				

[1] The data used to fit the result in this table is the adjuestd reaction time. ICC: Intraclass Correlation Coefficient.
[2] Significant effects are in bold. The false discovery rate (FDR) method was used for adjusted p-values.

We constructed a linear mixed-effects model to examine the impact of the ADHD group and the Stroop subtests on the correct rate. The model included the interaction between the subtests and the ADHD group, along with covariates such as age, medication treatment, and handedness. Random variability among individuals was accounted for by incorporating a random intercept for Patient ID. The basic formula used for modeling is shown below:

```
Correct Rate ~ Subtests*ADHD Group+ Age+ Medication Treatment
+ Left handedness+ (1|Patient ID)
```

Raction Time

In reaction time, linear mixed effects models account for stimulus presentation time, ADHD group, age, medication use, the correctness of response, and which hand (left or right) responded to each stimulus. For the general effects of all variables, the basic formula used as:

```
Reaction Times ~ 1 +  ADHD Group+ Subtests+ Stimulus Presentation Time
```

```
+ Age + Medication Treatment + Response hand + Correct answer
+ (1+ Subtests|Patient ID)
```

The results of the models in unadjusted and adjusted reaction times were shown in Table S2 and S3.

Table S2. Coefficients, standard errors, t-values, and p-values for the linear mixed effect model for **unadjusted** reaction time

**Variable**	**Coefficient (%95CI)**	**SE**	**t**	***p-value***[2]	***Adj.p***[2]
Intercept	1180.57 (1043.63, 1317.51)	69.85	16.90	**<0.001**	**<0.001**
Age	-86.65 (-112.25, -61.04)	13.06	-6.63	**<0.001**	**<0.001**
ADHD-I	100.22 (-41.82, 242.25)	72.45	1.38	0.16	0.17
Correct Answer	357.87 (272.87, 442.87)	43.36	8.25	**<0.001**	**<0.001**
Color Test	-106.17 (-190.14, -22.20)	42.83	-2.48	**0.02**	**0.03**
Color-Word Test	100.92 (-1.08, 202.93)	52.03	1.94	0.06	0.07
Medication Treatment	135.26 (-0.72, 271.24)	69.36	1.95	**0.05**	0.06
Response Hand (Right)	75.45 (38.34, 112.57)	18.93	3.99	**<0.001**	**<0.001**
Stimulus Presentation Time	-4.46 (-5.80, -3.11)	0.69	-6.49	**<0.001**	**<0.001**
**ICC** [1]					
Adjusted ICC	0.265				
Unadjusted ICC	0.238				

[1] ICC: Intraclass Correlation Coefficient.
[2] Significant effects and interactions are shown in bold. The false discovery rate (FDR) method was used for adjusted p-values.

Table S3. Coefficients, standard errors, t-values, and p-values for the linear mixed effect model for **adjusted** reaction time

**Variable**	**Coefficient (%95CI)**[1]	**SE**	**t**	***p-value***[2]	***Adj.p***[2]
Intercept	569.73 (489.30, 675.68)	47.53	12.25	**<0.001**	**<0.001**
Age	-37.31 (-50.25, -24.36)	6.60	-5.65	**<0.001**	**<0.001**
ADHD-I	50.19 (-21.45, 121.84)	36.55	1.37	0.17	0.18
Correct Answer	310.13 (258.04, 362.22)	26.57	11.67	**<0.001**	**<0.001**
Color Test	-130.64 (-172.70, -88.59)	21.45	-6.09	**<0.001**	**<0.001**
Color-Word Test	97.58 (-13.91, 209.07)	56.87	1.72	0.09	0.11
Medication Treatment	91.86 (1.68, 182.04)	46.00	2.00	**0.05**	0.07
Response Hand (Right)	23.22 (-1.47, 47.91)	12.60	1.84	0.07	0.07
Stimulus Presentation Time	-3.47 (-4.37, -2.58)	0.46	-7.63	**<0.001**	**<0.001**
**ICC**					
Adjusted ICC	0.137				
Unadjusted ICC	0.124				

[1] The equations of the models are presented in the appendix. ICC: Intraclass Correlation Coefficient.
[2] Significant effects and interactions are shown in bold. The false discovery rate (FDR) method was used for adjusted p-values.

For more information, a comprehensive analysis was performed using a series of linear mixed-effects models to fit the full model, including interaction terms with the two ADHD groups. Here is the formula:

```
Reaction Times ~ 1 + ADHD Group*Stroop Subtests +
ADHD Group*Stimulus Presentation Time + ADHD Group*Age
+ ADHD Group*Medication Treatment + ADHD Group*Response hand
+ ADHD Group*Correct answer
+ (1 + Stimulus Presentation Time|Patient ID)
+ (1+ Stroop Subtests|Patient ID)
```

For the interaction of ADHD groups with other variables in each subtest, here is the formula:

```
Reaction Times ~ 1 +  ADHD Group:Stimulus Presentation Time
+ ADHD Group:Age + ADHD Group:Medication Treatment
+ ADHD Group:Response hand + ADHD Group: Correct answer
+ (1 + Stimulus Presentation Time | Patient ID)
```

Extraneous Movement Score (EMS)

The extraneous movement score (EMS) captures the hyperactivity level of participants during the response process. Age is an essential factor for EMS. Figure S1 displays the violin plot and boxplot of the extraneous movement score in different ADHD and age groups (age $>$ 10 years and age $\leq$ 10 years) to provide a clear and unambiguous representation of the effect of age on this variable. The basic model used in the test showed below:

```
EMS~1+ Subtests+ Stimulus Presentation Time+ ADHD Group
+ Response hand+ Age+ Medication status
+ Correct answer+ Show Color+ +(1|ID)
```

The results showed in Table **??**, and the model within the "Show Color" as random slope was:

```
EMS~1+ Subtests+ Stimulus Presentation Time
+ ADHD Group+ Response hand+ Age+ Medication status
+ Correct answer+ Show Color+ +(1+ Show Color|ID)
```

Table S4. Coefficients, standard errors, t-values, and p-values for extraneous movement score

**Variable**	**Coefficient (%95CI)**[1]	**SE**	**t**	***p-value***[2]	***Adj.p***[2]
Intercept	4.52 (3.56, 5.48)	0.49	9.24	**<0.001**	**<0.001**
Age	-0.18 (-0.25, -0.11)	0.04	-4.91	**<0.001**	**<0.001**
ADHD-I	0.19 (-0.22, 0.61)	0.21	0.91	0.36	0.37
Word test	-0.17 (-0.26, -0.08)	0.05	-3.58	**<0.001**	**0.001**
Color test	-0.14 (-0.23, -0.05)	0.05	-3.09	**0.002**	**0.002**
Correct Answer	0.28 (0.10, 0.45)	0.09	3.13	**0.002**	**0.002**
Show Color(Blue)	-0.38 (-0.55, -0.21)	0.09	-4.46	**<0.001**	**<0.001**
Show Color(Green)	-0.46 (-0.58, -0.33)	0.06	-7.16	**<0.001**	**<0.001**
Response Hand (Right)	0.32 (0.21, 0.43)	0.06	5.77	**<0.001**	**<0.001**
Medication Treatment	0.55 (0.14, 0.96)	0.21	2.65	**0.01**	**0.01**
Stimulus Presentation Time	0.03 (0.02, 0.04)	0.01	4.14	**<0.001**	**<0.001**
**ICC**					
Adjusted ICC	0.281				
Unadjusted ICC	0.248				

1. The equations of the models are presented in the appendix. The extraneous movement score was logarithmically transformed. ICC: Intraclass Correlation Coefficient.
2. Significant effects and interactions are in bold. The false discovery rate (FDR) method was used for adjusted p-values.

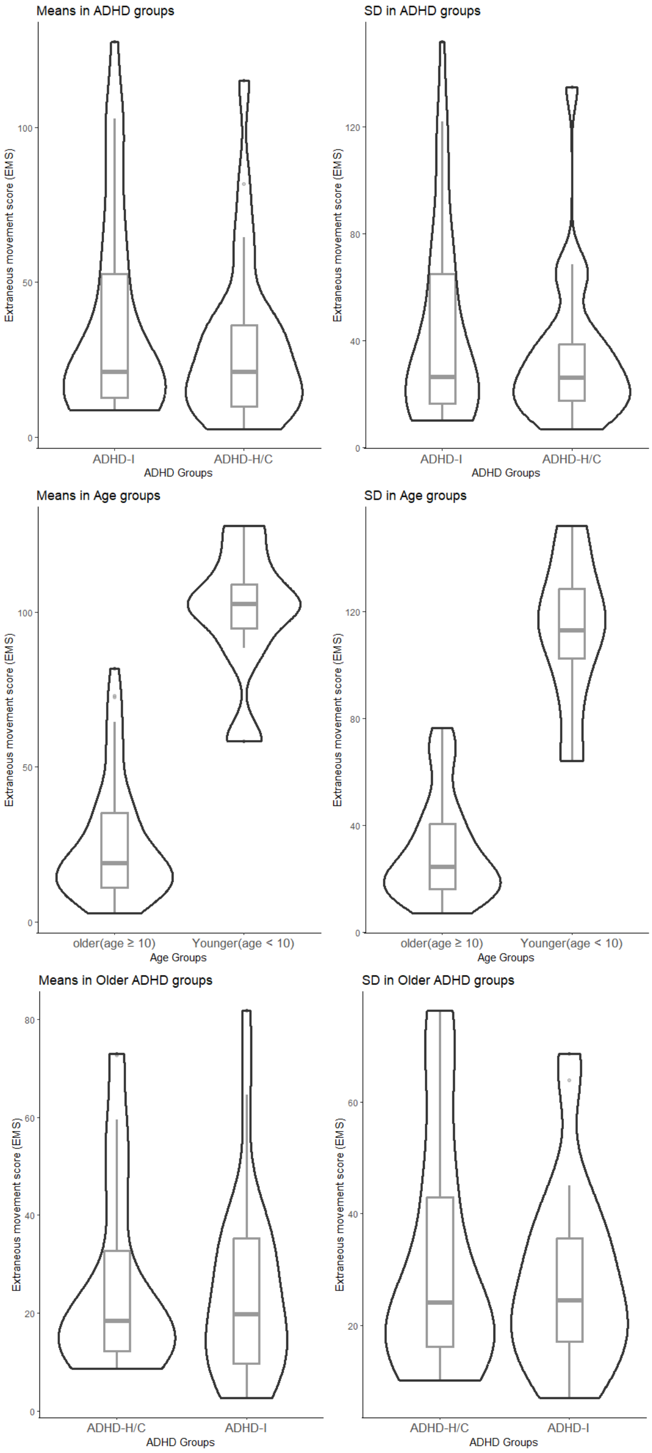

Figure S1. violinplot and boxplot of the extraneous movement score in different ADHD and age groups
